# Development and initial evaluation of the SCI-FI/AT

**DOI:** 10.1179/2045772315Y.0000000003

**Published:** 2015-05

**Authors:** Alan M. Jette, Mary D. Slavin, Pengsheng Ni, Pamela A. Kisala, David S. Tulsky, Allen W. Heinemann, Susie Charlifue, Denise G. Tate, Denise Fyffe, Leslie Morse, Ralph Marino, Ian Smith, Steve Williams

**Affiliations:** 1 New England Regional Spinal Cord Injury Center, Boston, MA, USA; 2 Department of Physical Therapy, University of Delaware, Newark, DE, USA; 3 Kessler Foundation, West Orange, NJ, USA; 4 Rehabilitation Institute of Chicago, Chicago, IL, USA; 5 The Rocky Mountain Regional Spinal Injury System, Englewood, CO, USA; 6 Department of Physical Medicine and Rehabilitation, University of Michigan, Ann Arbor, MI, USA; 7 Department of Physical Medicine and Rehabilitation, Rutgers NJ Medical School, Newark, NJ, USA; 8 Spaulding Rehabilitation Hospital, Boston, MA, USA; 9 Sidney Kimmel Medical College at Thomas Jefferson University, Philadelphia, PA, USA; 10 University of Pittsburgh Medical Centers, Pittsburgh, PA, USA; 11 University of Louisville, Louisville, KY, USA

**Keywords:** Outcome assessment (health care), Psychometrics, Quality of Life, Rehabilitation, Spinal cord injuries

## Abstract

**Objectives:**

To describe the domain structure and calibration of the Spinal Cord Injury Functional Index for samples using Assistive Technology (SCI-FI/AT) and report the initial psychometric properties of each domain.

**Design:**

Cross sectional survey followed by computerized adaptive test (CAT) simulations.

**Setting:**

Inpatient and community settings.

**Participants:**

A sample of 460 adults with traumatic spinal cord injury (SCI) stratified by level of injury, completeness of injury, and time since injury.

**Interventions:**

None

**Main outcome measure:**

SCI-FI/AT

**Results:**

Confirmatory factor analysis (CFA) and Item response theory (IRT) analyses identified 4 unidimensional SCI-FI/AT domains: Basic Mobility (41 items) Self-care (71 items), Fine Motor Function (35 items), and Ambulation (29 items). High correlations of full item banks with 10-item simulated CATs indicated high accuracy of each CAT in estimating a person's function, and there was high measurement reliability for the simulated CAT scales compared with the full item bank. SCI-FI/AT item difficulties in the domains of Self-care, Fine Motor Function, and Ambulation were less difficult than the same items in the original SCI-FI item banks.

**Conclusion:**

With the development of the SCI-FI/AT, clinicians and investigators have available multidimensional assessment scales that evaluate function for users of AT to complement the scales available in the original SCI-FI.

## Introduction

Functional performance is a critical outcome of many clinical trials. Generic functional outcome measures developed for the general population often lack the sensitivity needed to detect meaningful differences in the spinal cord injury (SCI) population, and they also contain irrelevant and sometimes offensive questions that lack validity in this population.^1^ Patient or condition-specific instruments often lack the scope of coverage that limits their utility in broad, heterogeneous samples of patients with a SCI.^2–5^

In recognition of these limitations, the Spinal Cord Injury Functional Index (SCI-FI) was developed by a team from seven SCI Model Systems Centers. This successful collaboration resulted in a new comprehensive activity limitations outcome instrument (called SCI-FI) that offers unique advantages over outcome measures developed using classical test theory methods. The content of the SCI-FI is based upon an extensive review of the literature, input from patients with SCI and clinician focus groups, and grounded within the International Classification of Function, Disability, and Health (ICF) framework.^6^ An extensive item pool was developed across several hypothesized functional domains and administered to 855 adults with a traumatic SCI. Investigators applied factor analysis and item response theory (IRT) methods to identify five distinct functional domains (Ambulation, Basic Mobility, Fine Motor Function, Self-care and Wheelchair Mobility). Computer adaptive test (CAT) and short form methodologies were employed to allow the SCI-FI to achieve measurement precision while reducing participant burden.^7–9^

There is a growing body of evidence to support the psychometric properties^10^ of the SCI-FI item banks.^10–12^ A SCI-FI pilot study in 269 adults with recent SCI demonstrated excellent responsiveness to change during the first post-injury year. The SCI-FI achieved excellent 1–2 week test-retest reliability, with Pearson's *r* values ranging from 0.92 (Wheelchair Mobility) to 0.96 (Self-care).^10^

Assessing the impact of Assistive Technology (AT) on a person's function is a significant problem that must be addressed when developing a rehabilitation outcome measure. When ability to do an activity is scored without regard to use of AT, the functional score may improve without a concomitant change in ability, thus weakening the relationship between impairment and activity.^13^ Unfortunately, the rehabilitation field lacks a consistent approach for assessing function when AT is used. Rust and Smith analyzed 100 rehabilitation measures and categorized how these measures addressed AT use.^14^ AT use was ignored in some measures, while other measures raised or lowered functional scores for items when the person used AT. Current SCI functional measures, such as the Spinal Cord Independence Measure, Quadriplegic Index of Function and the Functional Independence Measure account for the impact of AT using a hybrid approach where the use of AT is considered for some, but not all items. For items that consider AT, the item score is lower when AT is used than when it is not. However, because of the inconsistent consideration of AT in item scores, the summary scores derived from these measures do not fully reflect the impact of AT on a person's ability to function.^4,5,15^

The original SCI-FI banks contain few items in the calibrated item pool that measure a person's performance using AT. Outside of the wheelchair mobility scale, only 17 of 219 items calibrated in the SCI-FI item pool involve the use of AT. Moreover, the instructions to examinees who complete the SCI-FI item banks instruct participants to rate each item on their ability to perform the task without the use of AT or help from another person.

Given the paucity of AT related items and the focus on completion of tasks without devices, the original SCI-FI is likely to be less sensitive to how people perform activities in their usual settings when they would typically use AT to complete a task. The SCI-FI banks are also not likely to be precise and sensitive to clinically relevant change for interventions where the focus is on function using usual and customary AT. For this reason, we developed the SCI-FI/AT to provide supplemental item content that is most appropriate for AT users.

In this manuscript, we present analysis of the structure of the SCI-FI/AT item banks, describe the calibration and development of the SCI-FI/AT items and scales in each identified domain, discuss the initial psychometric properties of the SCI-FI/AT item banks, and compare the SCI-FI/AT item level difficulties with parallel item content in the original SCI-FI.

## Methods

### Sample

This SCI-FI/AT sample included 460 participants with a diagnosis of traumatic SCI recruited from 9 national SCI Model Systems programs. Eligibility criteria included age of 18 or older and the ability to speak and understand English. To best test functional performance with AT, the sample was stratified to include approximately equal numbers of individuals with the following characteristics: level of injury (paraplegia vs. tetraplegia), severity of injury (complete vs. incomplete) and time since injury (<1 year, 1–3 years, >3 years). The sample was representative of the key demographic variables reported by the National Spinal Cord Injury Statistical Center (NSCISC).^16^ The original SCI-FI sample is described in detail elsewhere.^7,8^

### Item bank development

We reviewed the previously calibrated SCI-FI item banks to retain as many items as possible from the original SCI-FI version so that similar items would be administered in the SCI-FI/AT, but with different instructions about the use of AT. The original SCI-FI items in each item bank were reviewed by two investigators (Alan M. Jette, Mary D. Slavin) to determine if items could be retained in SCI-FI/AT. This review identified original SCI-FI items that included use of a specific type of AT or human assistance. These items were removed, as they were inappropriate for SCI-FI/AT instructions which ask participants to select a response based on their ability “without help from another person, but using the equipment or devices they normally use.” After inappropriate items were removed, the SCI-FI/AT item content for each domain was again reviewed to ensure that all important functional activities assessed in the original SCI-FI were also included in SCI-FI/AT. It was noted that items related to bathing and skin inspection had been removed from the Self Care domain, so we wrote two new items using the relevant item content from the original version, but removing the reference to AT.

The final item pools administered in the SCI-FI/AT calibration study included: 47 Basic Mobility items, 35 Fine Motor Function items, 78 Self-care items, and 30 Ambulation items. The Basic Mobility item bank contains items about an individual's ability to carry out activities involving changing and maintaining body position, transfers, moving and carrying objects and moving around in different locations. The Ambulation items assess the ability to engage in walking activities in different locations that vary based on speed, time and condition and the ability to manage stairs under different conditions.

### Data collection procedures

All study personnel completed a 2-hour training session and successfully completed a certification program. Each site received a detailed manual of procedures and data collectors participated in bi-weekly quality control calls. To replicate the procedure used in the original SCI-FI calibration study, all SCI-FI/AT items were presented in interview format, either over the phone or in-person. Demographic information (e.g. age, sex, ethnicity, race), descriptors related to the spinal cord injury (e.g. date of injury, mechanism of injury, level of lesion, and severity) and responses to screener questions to select appropriate sets of supplemental items (e.g. mobility status, living situation, use of bowel and bladder program) were collected for all participants. Color-coded response cards were used to direct participants to the appropriate response set for each item or set of items. In contrast to the SCI-FI instructions, SCI-FI/AT participants were asked to select the response that best describes their current ability to do each activity *without help from another person, but using the equipment or devices they normally use*. Participants could skip an item if they were not able to determine a correct response. The data collection system automatically added supplemental items based on each participant's responses to screener questions (e.g. items about walking were only administered to ambulatory participants).

### Data analytic procedures

We employed confirmatory factor analysis (CFA) to examine the unidimensionality in each domain based on weighted least squares means and variance adjusted (WLSMV) estimation. We used the χ^2^ test and multiple fit indices (e.g. Tucker-Lewis Index, Comparative Fit Index)^17,18^ to assess the model fit. We also examined local item dependence (LID)^19^ by looking at the residual correlation matrix output from Mplus.^20^ Any item pair with an absolute residual correlation greater than 0.2^21^ was considered to exhibit unacceptable LID and one of the items was removed from the item pool.

Prior to finalizing the item banks, we used a three-step procedure to examine Differential Item Functioning (DIF) in group variables (sex and diagnosis—tetraplegia and paraplegia). First, each item response was fitted to two ordinal logistic regression models. The first model included the raw summed score as the single independent variable, while the second model included the three independent variables of raw summed score, group variable, and interaction between the raw summed score and group variable. Any item that exhibited an R-squared change of greater than 0.02^22^ between the two models was flagged for potential DIF. For each flagged item, we examined item category frequency distributions across subgroups (i.e. female vs. male or paraplegia vs. tetraplegia); if any subgroup sample size was less than 100 or if an item category's percentage of responses was greater than 90% (i.e. a dominating response category), this item's calibration was rejected for this subgroup.^23,24^ Next, we set the non-DIF items' parameters equal across subgroups and calibrated the DIF items separately in each subgroup based on a two-group IRT model. We then examined the item parameter equality based on Wald's χ^2^ test^25^ and used the Benjamini–Hochberg (BH) procedure^26^ to adjust the P value. Finally, we applied the weighted Area Between the expected score Curves (wABC) to examine the DIF impact.^27^ The wABC was calculated by integrating the absolute difference between the expected core functions of reference and focal samples over the latent distribution. Any DIF item with wABC > 0.4^27^ was treated as demonstrating DIF. Since our goal was to retain as many items as possible, we either calibrated the DIF item separately in relevant subgroups if the sample size in each subgroup was greater than 100 and there was no dominating response category, or only calibrated an item in one subgroup with the expectation that these items will only be administered in this subgroup in the future. We wanted to retain the maximum number of items and obtain stable item calibrations. Therefore, we adopted the least restricted requirement for Rasch models (100 subjects). Most of the subgroup sample sizes were around 200 in the examined items, which is the minimum number requirement in 2-parameter model. The analytic model was successfully converged, items showed a reasonable hierarchical difficulty order and we examined the item fit based on calibrated item parameters.

**Figure 1:**
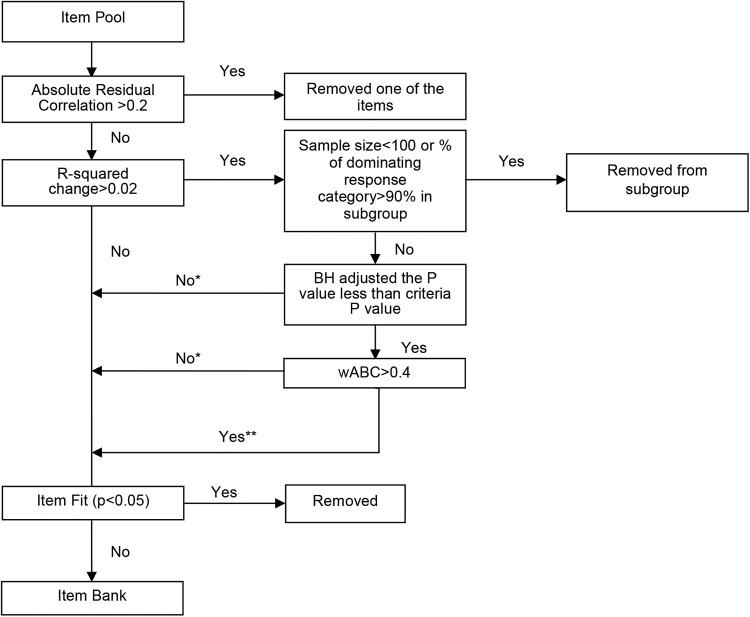
Algorithms for Statistical Procedures used to construct Item Pool. *Treated as the same item in subgroup; **treated as different item in subgroup.

Each item bank was calibrated with the Graded Response Model^28^ as implemented in IRTPRO. The Pearson χ^2^ test, which examines the discrepancy between the expected and actual number of subjects in each category at each summed score level (S-X^2^ Test) was used to assess item fit. An item with a test statistic P value less than 0.05 was eliminated from the final item bank (Fig. 1).

Subject scores on the final item banks were estimated with weighted maximum likelihood estimation. We also conducted real data simulations in SAS 9.13 (SAS Institute Inc., Cary, NC, USA) to estimate person scores on 10-item fixed-length computerized adaptive tests. To compare the differences in item parameters between common items to the SCI-FI and SCI-FI/AT, we identified 41, 68, 33 and 29 common items between two versions in Basic Mobility, Self-care, Fine Motor and Ambulation domains, respectively. One item in basic mobility, 6 items in Self-care and 5 items in Fine Motor Function calibrated separately in subgroups in SCI-FI/AT. We eliminated those items in an equality test. To test item parameter invariance in the evaluation of IRT-based DIF, we would need to have the full covariance matrix of the item parameter estimations from both versions. However, SCI-FI was calibrated in PARSCALE which doesn't provide such information. Based on the similarity of sample characteristic across two calibration samples, we re-calibrated the common items across both versions at the same time using IRTPRO (in the Ambulation scale, we only looked at the item parameters when we calibrated the items based on the subjects who walk some or all the time). We assessed invariance of the paired common items by using a Wald χ^2^ test (BH adjusted P value) and plotted the paired common item difficulty in each domain. Item difficulty is defined as the average value of threshold parameters in the Graded Response Model. The threshold parameter for response category x represents the point on the underlying concept at which the subject will have 50% probability of endorsing this category or higher. All the analyses were done in Mplus 6.0,^20^ IRTPRO 2.10,^29^ and SAS 9.13.^30^

## Results

 Table 1 displays the background characteristics of the SCI-FI and SCI-FI/AT samples. The demographic makeup of the two samples was quite similar: just over half of each sample with tetraplegia, and in both samples the average age was 43 years and over three quarters of participants were male and living at home. One notable difference is that there were more participants of Hispanic origin in the SCI-FI (11%) sample as compared with the SCI-FI/AT sample (5%).

**Table 1:** Background Characteristic of original SCI-FI and SCI-FI/AT Calibration Samples

Characteristic	Original SCI-FI (*N* = 855) Mean(SD) or %	SCI-FI/AT (*N* = 460) Mean(SD) or %	Statistical test between two samples* P value
Current age(y)	43.0(15.3)	43.1(14.8)	0.909
Age at injury(y)	36.0(15.7)	36.6(15.4)	0.506
Time since injury(y)	7.0(9.3)	6.5(8.9)	0.346
Ethnicity			
Hispanic	11.3	5.2	<0.001
Sex			
Male	77.0	81.1	0.092
Race			
White	70.4	73.7	
Black	17.3	20.7	
Other	12.3	3.7	<0.001
Level of injury (Diagnosis)			
Paraplegia	45.6	46.5	
Tetraplegia	54.4	53.5	0.772
Level of injury (ASIA-Confirmed)			
Complete	46.0	46.2	
Incomplete	54.0	46.0	0.168

*Two-group *t* test used in continuous variables, χ^2^ test used in categorical variables.

 Table 2 presents the CFA results along with goodness-of-fit indices for unidimensional CFA models of the SCI-FI/AT item banks. Results replicate the original SCI-FI structure with acceptable fit statistics for distinct domains of Basic Mobility, Self-care, Fine Motor Function, and Ambulation.

**Table 2:** Goodness-of-fit Indices for Unidimensional CFAs for the SCI-FI/AT final Item Banks*

Subscales	No. of Items	χ^2^	df	χ^2^/df	CFI	TLI	RMSEA
Basic Mobility	41	2957.7	779	3.7	0.97	0.97	0.07
Self-care (women)	66	5067.7	2079	2.4	0.99	0.99	0.05
Self-care (men)	68	5261	2210	2.3	0.99	0.99	0.05
Fine Motor Function	35	1311.1	560	2.3	0.99	0.99	0.05
Ambulation	29	585	377	1.5	0.99	0.99	0.03

*The Self-care bank includes a total of 71 items: for women, 66 items are included, for men, 68 items are included.

Note: CFI, Comparative Fit Index; TLI, Tucker-Lewis Index; RMSEA, Root Mean Square Error of Approximation.

As seen in Table 3, not all initial items were retained in the final calibrated item banks. Of the 190 items included in the SCI-FI/AT study, 176 distinct items were retained and calibrated with IRT analysis. One misfitting item and 9 LID items were removed from the final item bank. No items exhibited DIF for sex. However, 17 identified items exhibited DIF by level of injury. Of these, 5 items were removed and the remaining 12 items with DIF by level of injury were calibrated separately by level of injury and retained in the final item banks. We ended up with 41 items in the Basic Mobility domain, 71 in the Self-care domain, 35 in the Fine Motor Function domain, and 29 in the Ambulation domain.

**Table 3:** SCI-FI/AT Item Bank Refinement

Domain	Initial	DIF			Items Removed			Final item bank
		Items displaying DIF*	Item(s) calibrated in paraplegia and tetraplegia separately	Item(s) calibrated only in tetraplegia	DIF	Local dependent	Misfit items	
Basic Mobility	47	2	1	0	1	5	0	41
Self-care	78	9	1	5	3	4	0	71
Fine Motor Function	35	5	1	4	0	0	1	35**
Ambulation	30	1	0	0	1	0	0	29
Total items	190	17	3	9	5	9	1	176

*There is no sex DIF, the number in the tables are the items identified with diagnostic DIF (i.e. paraplegia, tetraplegia).

**The misfit item was the item calibrated separately in paraplegia and tetraplegia, so the number of items in Fine Motor Function item bank is 35.

Note: DIF, Differential item functioning.

**Figure 2:**
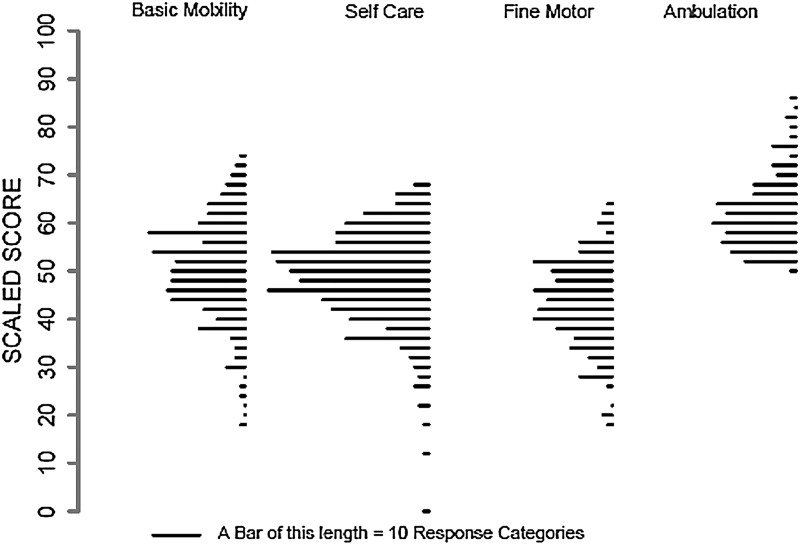
Distribution of the SCI-FI/AT items/calibrations for each content domain.

As seen in Table 4, the calibration dataset was used to compare the accuracy and breadth of coverage of the full bank and a 10-item simulated CAT for each SCI-FI/AT domain by level of injury. The correlation of the 10-item CATs with the full item bank exceeded 0.97 for all 4 SCI-FI/AT domains across both paraplegia and tetraplegia. For those with tetraplegia, there were minimal ceiling and floor effects for all 4 functional domains. For those with paraplegia, there were minimal to no floor effects seen for all domains and a minimal ceiling effect for the Basic Mobility domain. As expected, 35% or more of the sample with paraplegia were at the ceiling of the Fine Motor Function scale and 15–18% of this sample was at the ceiling of the Self-care function scale. Fig. 2, which displays the distribution of response categories/items for each functional domain, illustrates the relative paucity of response categories/items above 2 standard deviations from the mean for the SCI-FI/AT domains of Fine Motor Function and Self-care which correlates with the ceiling effects seen in participants with paraplegia.

**Table 4:** Accuracy, Breath of Coverage for a SCI-FI/AT 10-item CAT and Full Item Bank for Each Content Domain by Neurologic Level

Domain	Mode	N	Tetraplegia					N	Paraplegia				
			Accuracy**	Mean ± SD	Range	%Ceiling	%Floor		Accuracy	Mean ± SD	Range	%Ceiling	%Floor
Basic Mobility	10-item CAT	246	0.98 (0.97,0.98)	46.7 ± 10.2	23.4–73.7	2	5.3	213	0.97 (0.96,0.97)	56.4 ± 6.6	36.1–73.7	2.4	0
	Full item bank	246	–	46.5 ± 10.1	21.1–76.8	0	4.5	213	–	56.4 ± 6.5	35.9–75.4	1.4	0
Self-care	10-item CAT	246	0.98 (0.97,0.98)	46.6 ± 11.6	6–69	3.3	5.3	213	0.97 (0.96,0.98)	60.3 ± 6.5	29.3–69.3	18.3	0
	Full item bank	246	–	46.5 ± 11.4	6.5–70.1	1.6	1.6	213	–	60.7 ± 6.6	30.9–70.4	15.0	0
Fine Motor Function	10-item CAT	245	0.98 (0.98,0.99)	42.6 ± 9.8	19.8–64.1	2.9	4.9	212	0.98 (0.98,0.99)	57.5 ± 5.3	30.2–62.4	36.8	0
	Full item bank	245	–	42.4 ± 10.1	17.8–64.5	1.6	4.5	212	–	57.7 ± 5.4	30–62.7	35.9	0
Ambulation*	10-item CAT	70	0.98 (0.97,0.99)	61.1 ± 5.6	48.7–79.1	0	1.4	50	0.99 (0.99,0.99)	58.9 ± 6.5	48.7–83.2	2.0	6
	Full item bank	70	–	60.9 ± 5.5	48.1–79.2	0	1.4	50	–	58.9 ± 6.5	48.1–83.1	2.0	6

*Limited to participants who walk some or all of the time.

**Intraclass Correlation Coefficient (95%CI) between 10-item CAT scores and full item bank scores.

Note: CAT, Computer Adaptive Test.

 Table 5 presents the reliability of each domain scale for the simulated 10-item CAT and full item bank. As these data illustrate, 75–98% of subjects achieved reliability >0.90 in each content domain.

**Table 5:** Number (%) of subjects with reliability >0.9 for 10-item CATs and the full item bank for each content domain

	Basic mobility	Self-care	Fine Motor Function	Ambulation
10-Item CAT	409(89.11%)	391(85.19%)	345(75.49%)	118(98.33%)
Full Item Bank	435(94.77%)	411(89.54%)	361(78.99%)	118(98.33%)

Note: CAT, Computer Adaptive Test.

**Figure 3:**
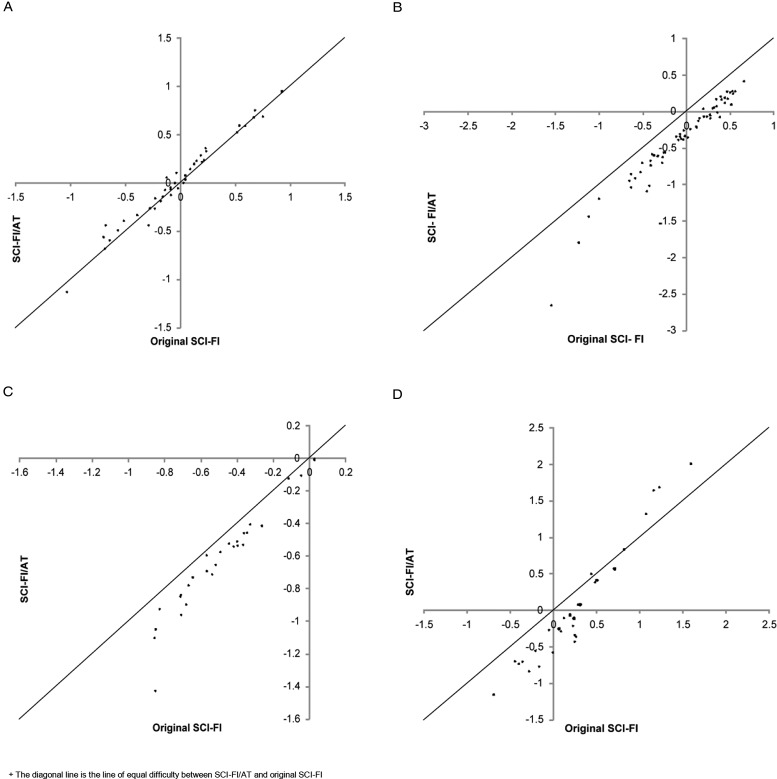
Comparison of Item Difficulty in SCI-FI versus SCI-FI/AT for each content domain. (A) Basic Mobility. (B) Self-care. (C) Fine Motor Function. (D) Ambulation.

Finally, Fig. 3A–D presents a comparison of the level of difficulty of common items in SCI-FI versus SCI-FI/AT for each content domain. While there is little difference in item difficulty in the Basic Mobility domain scale (12.5%), for the most part, SCI-FI/AT items in the domains of Self-care (90.3%), Fine Motor Function (75.0%) and Ambulation (51.7%) were less difficult than the same items in SCI-FI, which is consistent with the DIF results.

## Discussion

The SCI-FI instruments are multidimensional activity limitation measures appropriate for persons with SCI, regardless of level or severity of injury. The original SCI-FI scales allow for the assessment of functional capacity in the domains of Basic Mobility, Ambulation, Self-care, and Fine Motor Function. With the development of the SCI-FI/AT, we now have available scales that evaluate a person's ability to function using AT in the areas of Basic Mobility, Self-care, Ambulation, and Fine Motor Function along with the previously calibrated Wheelchair domain. In this study, the SCI-FI/AT replicated the domain structure in the original SCI-FI instrument and revealed strong psychometric properties for all domain scales in a sample of adults with paraplegia or tetraplegia. High correlations of simulated 10-item CATs with full item banks indicated high accuracy of the CAT in estimating a person's underlying functional ability. Furthermore, there was high measurement reliability for the simulated CAT scales compared with the full item bank.

SCI clinical trials, efforts to enhance our understanding about the plasticity of the nervous system and potential for neurorecovery, and studies of the efficacy of current rehabilitation interventions require functional outcome instruments that are capable of evaluating not only a person's functional capability but also one's ability using whatever AT and accommodations that are employed by the patient to accomplish the desired activity.^31^ Although they are sometimes used interchangeably, clear distinctions exist between the concept of functional capacity and functional performance^32^ and these distinctions must be reflected in the development and selection of outcomes instruments.^13^ Capability refers to what a person can do without assistance while performance reflects what the person does in his or her environment using whatever means are available.^33^ For instance, studies examining the actual neurorecovery experienced by individuals or evaluating new physical therapy interventions designed to restore underlying functional capacity would benefit from the inclusion of SCI-FI banks. In contrast, the SCI-FI/AT would be a much more important measure and potentially more sensitive measure to include in trials of new types of adaptive equipment or of occupational therapy interventions designed to improve daily functioning at a constant level of functional capacity. Furthermore, the SCI-FI could be used to justify continued rehabilitation to payers who may require documentation of continued recovery of functional capacity (SCI-FI) or functional performance (SCI-FI/AT) as a requirement for continuity of care.

The CAT methodology for administering the SCI-FI assessments is ideally suited for the complexity faced when assessing functional abilities of those with SCI and overcomes many of the conceptual and methodological limitations with traditional measurement approaches.^2–5^^,^^15^ A CAT can be used to assess selective functional domains with a common metric and the existence of SCI-FI and SCI-FI/AT versions allows for the investigator or clinician to assess either functional capacity or functional performance, or both. Instead of using multiple traditional instruments, one can employ filter questions to select the appropriate domains and items that reflect a person's clinical and demographic background, thus avoiding inappropriate functional domains and items.

Although the SCI-FI now provides researchers and clinicians with the ability to assess functional performance across multiple domains for persons with SCI, there are several limitations to this study that should be noted. For those with paraplegia, the breadth of coverage in the Self-care and Fine Motor Function scales could be improved by including more challenging tasks in each item pool, thus reducing some of the ceiling effects that were observed in the calibration sample. Secondly, further psychometric analyses on new samples is needed to confirm the results reported in this study. In fact, a validation study of the SCI-FI/AT instrument is underway in an independent sample that will examine its validity and responsiveness to clinically meaningful change in comparison with the SCI-FI and legacy instruments. And finally, having more participants of Hispanic origin in the original SCI-FI sample (11%) as compared with the SCI-FI/AT sample (5%) could account for some of the difference in item difficulty that were observed across the two versions of SCI-FI.

## Conclusion

The findings from this study provide preliminary evidence that the SCI-FI/AT's domain structure is similar to the SCI-FI and revealed strong psychometric properties for all functional domain scales in a sample of adults with paraplegia or tetraplegia. High correlations of simulated 10-item CATs with full item banks indicated high accuracy of the CAT in estimating a person's functional ability and there was high measurement reliability for the simulated CAT scales compared with the full bank scores. SCI-FI/AT item difficulties in the domains of Self-care, Fine Motor Function, and Ambulation were less difficult than the same items in SCI-FI item banks. With the development of the SCI-FI/AT, we have available multidimensional assessment scales that evaluate function using AT in the areas of Basic Mobility, Self-care, Ambulation, Fine Motor Function, and Wheelchair Mobility to complement the domain scales already available in the SCI-FI.

## Disclaimer statements

**Contributors** No other contributors.

**Funding** This work was supported by US Department of Education, National Institute of Disability and Rehabilitation Research Grant Numbers: H133N120002, H133N060014, H133N110006, H133N110020, H133N110002, H133N110021, H133N110011, H133N110007, H133N110009.

**Conflicts of interest** None.

**Ethics approval** The Institutional Review Board at each site reviewed and approved this project.
